# Clinical Observations and Laboratory Experiments

**Published:** 1884-11

**Authors:** C. M. Wright

**Affiliations:** Cincinnati


### CLINICAL OBSERVATIONS AND LABORATORY EXPERIMENTS.

BY C. M. WRIGHT, D. D. S., CINCINNATI.

READ BEFORE THE AMERICAN DENTAL SOCIETY OF EUROPE, AT ITS MEETING AT VEVEY, SWITZERLAND, 1884.

*Dear Brethren:*—At this, the twelfth annual meeting of the American Dental Society of Europe, I cannot help being present as a ghost, or in spirit, never having failed to present some sort of an appearance each year since the first feeble beginning of the Society on the Rigi. Distance has not prevented me from presenting letters to the society at the last two meetings, and I shall deem it a privilege to be allowed to do so again.

The American Dental Society of Europe is to-day, perhaps, one of the most important dental organizations in the world. It does much and good work; its members have excellent opportunities for original observations, and have no personal axes to grind; its reports are fresh and full of interest; its papers are looked forward to by the dental world with eager expectation; it numbers many of the most distinguished men in the dental profession of the day in its list of members; dentists, distinguished either in the operative and clinical department, or in the experimental department as laboratory investigators, bring the results of their labors to your annual meetings, and you reap the benefit by seeing yourselves placed in the front rank of societies. As a school or university becomes distinguished on account of the importance of the work and the reputation of its professors, so do societies take rank according to the character and work of the members. Every member of the American Dental Society of Europe, no matter how modestly he may view his own work or attainments, has reason to be proud of his position as a member of a society that has taken the lead, through one of its members, in scientific laboratory investigations. Recently the editor of an able medical journal said to me: "The American dentists of Europe have among them the foremost investigators, by all odds, in your profession." I replied: "Yes, and among the members of that society are some of the foremost clinical observers of the day, too."

Schools, universities, and even nations obtain distinction in definite fields, at certain periods or generations. Germany, for instance, now has a reputation for minute pathological observations, and American students flock to the German universities to pick up the crumbs that fall from the laboratory tables, and progressive professors in American medical colleges spend their summer vacations in Germany, that they may visit certain laboratories and partially satisfy their cravings for the knowledge to be found there.

America is distinguished for the clinical knowledge and the aptness of her surgeons and dentists in practice. While residing in

Basel I often heard bright German medical students regret that New York was so far away, for if it were not, German students would flock to its medical schools to witness the great skill of its surgeons and operators.

A German dentist practicing in Southern Germany once remarked: "Yes! yes! the Germans have it *here* (pointing to his forehead), and the Americans have it *here* (working his fingers in the air). *I* am German, but I have been educated in America, so *I* have it *here, and here* (in head and in fingers)." I might remark that this combination forms an excellent alloy for the construction of large brass monuments, and may be made useful in the arts.

Which is the better reputation to have; the German or the American? Which will do the most good in the world? Which takes the shortest and best route to the goal? Can we discuss such questions as Laboratory Investigations *versus* Clinical Experience? It seems to me that there can be no question of *versus* between the two methods of searching for truth. The two *must* go hand in hand before truth (which lies in a well) can be reached. If laboratory investigation after careful experiments asserts that truth can be approached only with a copper-bottomed bucket and a galvanized wire for a rope, and clinical observation has time and again reached truth with an old oaken bucket and a pole, laboratory investigation must go over its experiments again, for evidently there has been a mistake in some of the steps.

If laboratory investigation says that copper amalgams do not exert any influence in saving teeth from caries, and clinical observation has found by years of trial that they do, and that when for a certain reason the dentist wishes to save a badly organized tooth in a bad position he puts a copper amalgam filling into its ragged cavity with a certainty of salvation in his mind, born of years of trial, certainly there must be an error somewhere; truth has not been reached. Either clinical observation has been careless, has not been strictly and scientifically conducted and therefore is not worthy the name, or laboratory investigation has made some mistake or been at fault in some of its observations. When the two agree we can feel assured that a scientific fact has been established, and we should throw up our hats and make a fourth of July celebration over it, no matter how our prejudices may have suffered.

Now, my dear brethren, I must admit that while highly pleased with one result of recent laboratory investigation, I have been slightly disappointed in another. From clinical observations extending over several years, and in the face of a prejudice and unbelief, I had become convinced that our tin and gold combination (advocated by dear Doctor Abbot, at the meeting of our Society in Geneva, in 1874) did possess *therapeutic* virtues aside from its purely mechanical property of perfectly stopping against septic influences.

I also had come to believe that base-plate gutta-percha, employed as a tooth stopping, had certain antiseptic or saving properties not possessed by other gutta-perchas; and I have been in the habit of classifying in my own mind copper amalgam, tin and gold, and red gutta-percha, as "therapeutic fillings." I could not give scientific reasons based either on chemistry or physiology, but from oft-repeated clinical experiments and from clinical observations I had faith in these fillings as possessing special saving qualities aside from any mere mechanical properties. I had come to have vague notions like these; first, perhaps the peculiar combination of the coloring matter in the base-plate gutta-percha, and the remarkable molecular changes that occur in the tin and gold combination when exposed to the heat and fluids of the mouth, and the chemical changes that take place in the oral cavity in copper and mercury amalgams, do produce permanent aseptic conditions in the tooth.

Or, second, that the same changes in these materials do cause just the right amount of healthy irritation to the protoplasm of the living tooth to stimulate it to protect itself by new deposits of less highly organized dentine. Garretson sustains me in the employing of copper in the cavity of a tooth, claiming that even a sprinkling of copper fillings in the bottom of a cavity tends to stimulate the pulp to throw out "secondary dentine."

Now, of course, these theories are of no value whatever, and are at the mercy of the intelligent laboratory investigator; but the clinical observations are the same. This, I think, is an important fact, and we must look to the laboratory for a correct scientific explanation, and then we may all feel that a truth has been revealed. The field of medicine is full of just such questions. In dentistry, as in medicine, laboratory investigation and clinical observation must agree.

Laboratory investigation must give good reasons for the facts of clinical observations, or the experiments must be gone over again; or clinical experiments must be repeated for proofs of the assumed facts.

Where can we, in our profession, have these things better established than in a society that so perfectly combines the head and the fingers as does the American Dental Society of Europe?

Brethren, the medical and dental worlds are "looking toward you" with great expectations and good wishes as you stand at the "bar" of the world, and I beg the privilege of looking toward every one of you, my old friends, as you sit at the final banquet and drink each other's health, before you say good-bye to Switzerland, the birthplace and cradle land of the American Dental Society of Europe.

